# Predicting Length of Stay among Patients Discharged from the Emergency Department—Using an Accelerated Failure Time Model

**DOI:** 10.1371/journal.pone.0165756

**Published:** 2017-01-20

**Authors:** Chung-Hsien Chaou, Hsiu-Hsi Chen, Shu-Hui Chang, Petrus Tang, Shin-Liang Pan, Amy Ming-Fang Yen, Te-Fa Chiu

**Affiliations:** 1 Department of Emergency Medicine, Chang Gung Memorial Hospital, Linkou and Chang Gung University College of Medicine, Taoyuan, Taiwan; 2 Institute of Epidemiology and Preventive Medicine, College of Public Health, National Taiwan University, Taipei, Taiwan; 3 Graduate Institute of Biomedical Sciences, College of Medicine, Chang Gung University, Taoyuan, Taiwan; 4 Department of Physical Medicine and Rehabilitation, National Taiwan University Hospital and National Taiwan University College of Medicine, Taipei, Taiwan; 5 School of Oral Hygiene, College of Oral Medicine, Taipei Medical University, Taipei, Taiwan; University College London, UNITED KINGDOM

## Abstract

**Background:**

Emergency department (ED) crowding continues to be an important health care issue in modern countries. Among the many crucial quality indicators for monitoring the throughput process, a patient’s length of stay (LOS) is considered the most important one since it is both the cause and the result of ED crowding. The aim of this study is to identify and quantify the influence of different patient-related or diagnostic activities-related factors on the ED LOS of discharged patients.

**Methods:**

This is a retrospective electronic data analysis. All patients who were discharged from the ED of a tertiary teaching hospital in 2013 were included. A multivariate accelerated failure time model was used to analyze the influence of the collected covariates on patient LOS.

**Results:**

A total of 106,206 patients were included for analysis with an overall medium ED LOS of 1.46 (interquartile range = 2.03) hours. Among them, 96% were discharged by a physician, 3.5% discharged against medical advice, 0.5% left without notice, and only 0.02% left without being seen by a physician. In the multivariate analysis, increased age (>80 vs <20, time ratio (TR) = 1.408, p<0.0001), higher acuity level (triage level I vs. level V, TR = 1.343, p<0.0001), transferred patients (TR = 1.350, p<0.0001), X-rays obtained (TR = 1.181, p<0.0001), CT scans obtained (TR = 1.515, p<0.0001), laboratory tests (TR = 2.654, p<0.0001), consultation provided (TR = 1.631, p<0.0001), observation provided (TR = 8.435, p<0.0001), critical condition declared (TR = 1.205, p<0.0001), day-shift arrival (TR = 1.223, p<0.0001), and an increased ED daily census (TR = 1.057, p<0.0001) lengthened the ED LOS with various effect sizes. On the other hand, male sex (TR = 0.982, p = 0.002), weekend arrival (TR = 0.928, p<0.0001), and adult non-trauma patients (compared with pediatric non-trauma, TR = 0.687, p<0.0001) were associated with shortened ED LOS. A prediction diagram was made accordingly and compared with the actual LOS.

**Conclusions:**

The influential factors on the ED LOS in discharged patients were identified and quantified in the current study. The model’s predicted ED LOS may provide useful information for physicians or patients to better anticipate an individual’s LOS and to help the administrative level plan its staffing policy.

## Introduction

Emergency department (ED) crowding is a worldwide issue in all health care systems and is associated with the increased incidence of several adverse outcomes [1–3]. Although the etiology of ED crowding is complicated, it can be divided into three aspects: the input, throughput, and output of ED patients [4, 5].

The input of patients (ED visits) has increased significantly over the past two decades [6, 7], and because modern EDs can diagnose and treat a much wider range of patients compared to 20 years ago, it is unlikely that the trend in patient visits will decline in the near future. The destination of patient output (disposition) is mostly either home or stay at hospital. The process of ED admission is often difficult and patients need to wait and receive treatment in the ED observation room. According to a recent study, the ED now accounts for more than one-half of hospital admissions [8]. However, the output blockage is not an issue that can easily be tackled by the ED alone. In order to balance admissions and discharges, a larger scale of planning and coordination may be needed. For instance, it may be necessary for hospital-level administration to distribute available beds according to patient flows, different specialties, staffing changes, and seasonal fluctuations [9–11].

Because patient input and output processes are often related to broader health care issues, the throughput process is therefore left to be the main focus for researchers of ED crowding [4]. An important indicator of the patient management process is the ED length of stay (LOS). ED LOS has been identified as a cause as well as a result of ED crowding [12, 13]. Analyzing the ED LOS of discharged patients is especially important, because in most hospitals, these patients make up more than half of all ED visits and do not have the output blockage problem which often occurred for admitted patients [6, 13]. A recent research found that LOS of non-admitted patients negatively correlated with ED quality and performance indicator [5]. Previous studies also reported some of the factors contributing to prolonged ED LOS [14, 15]; however, the effect of individual or environmental factors on ED LOS has not been quantified and compared. The aim of this study was to identify and quantify the influence of different patient-related or diagnostic activities-related factors on the ED LOS of discharged patients.

## Methods

### Study design

This was a retrospective analysis of the administrative database from Linko Chang-Gung Memorial Hospital (LCGMH). The study protocol, variables analyzed, and statistical methods were determined before the study was conducted. The study was approved by the Chang Gung Memorial Hospital institutional review board (1045309B) and was exempt from the requirement of obtaining informed consent.

### Study setting and population

The study was conducted in the ED of LCGMH, a tertiary medical center and teaching hospital with a 3,600-bed capacity and an annual ED visit of approximately 150,000 patients. LCGMH is also a level I trauma center with all surgical subspecialties available 24 hours a day. The LCGMH ED contains one computer tomography (CT) examination room and two X-ray rooms within the ED area. Patients originate from the area of northern Taiwan and come in with general emergency complaints. The inclusion criterion was all patients who visited the ED of LCGMH and had been discharged from the ED from January 2013 to December 2013. These patients included those who left before being approached by an ED physician (left without being seen), those who were discharged by the ED physician after management completed, those who insisted to leave despite doctors suggested otherwise (left against medical advice), and those who left without notice. Patients with missing registration times or leaving times were excluded from the analysis.

The LCGMH ED contains a fast-track system for non-urgent medical patients. These generally include all adult non-trauma patients who were ambulatory (or at least could wait in a wheelchair) and were triaged as levels III to V. The fast-track system opens at eight in the morning and closes an hour after midnight. During these open hours, several urgent clinics will be in operation, each containing an emergency physician, an ED nurse, an examination bed, and urgent diagnostic tools, such as electrocardiograms (ECG) and bedside sonography machines. The number of urgent clinics ranges from one to three depending on average daily patient flow.

### Data collection

All data were drawn from the hospital’s administrative database. The time variables included triage time recorded by the triage nurse, physician time recorded by the computer when the first primary ED physician approached the patient, and time leaving the hospital recorded by the registration counter. A patient’s ED LOS was defined by the time from registration to leaving. The triage level was assigned by a specialized triage nurse using the five-level Taiwan Triage Acuity System (TTAS). Patients were divided into three general categories by the triage nurse: adult non-trauma, pediatric non-trauma, and trauma patients. The demographic variables collected included patient age and gender. The disease- and acuity-associated variables included patient category, triage level, whether the patient was transferred from another hospital, whether an admission or observation order was prescribed, and whether a critical status was announced. The decision of admission or observation was decided by the primary ED physician after discussed with patients and their family members. We also documented whether X-rays, CT exams, laboratory exams, consultations, and/or electrocardiograms were provided. The environmental variables included whether the patient came in during the weekend, the shift during which the patient arrived, and the total ED daily census in the same day. The ED daily census was incorporated as a binary variable with a cutoff point of 558 patients; this is the 95^th^ percentile of the study ED’s daily census.

### Statistical analysis

The primary outcome was the ED LOS of patients who were discharged from the ED. In descriptive analysis, a normality test was performed for continuous variables. The median and interquartile ranges (IQR) were used to describe the central tendency and the spread of data obviously deviating from the normal distribution. For event time analysis, the Kaplain–Meier method was used to construct an overall survival plot and plotted between strata. The log-rank test was used to compare the difference in survival curves between strata.

For the multivariate analysis of the influence of the collected variables, an accelerated failure time (AFT) model was used. The AFT model is a type of survival analysis that directly models the length of stay as a function of a constellation of factors [16]. The effect size of each factor on LOS is evaluated by regression coefficient (b). Taking the exponential of regression coefficient (b), *exp*[*b*_*i*_] are referred to as time ratios (TRs). A TR less than 1 indicates that the LOS is shortened, and a TR greater than 1 implies that the LOS is lengthened [17]. The model was further used to predict individual LOS based on personal influential factors. The predicted LOS was compared with the observed LOS by life-table method. Goodness of fit was also assessed using log-rank test. All analyses were performed using SAS statistical software version 9.3 (SAS Institute Inc., Cary, NC) [18]. A p value of less than 0.05 was considered statistically significant.

## Results

### Demographic results of included patients

In total, 106,206 patients and their data were included in the study. The mean age of the study population was 35.9 (standard deviation [SD] = 25.7) years, and 52.9% were male. The proportions of patients in different categories were 55.7%, 24.9%, and 19.4% for adult non-trauma, pediatric non-trauma, and trauma patients, respectively. Among five acuity levels, triage level III (63.7%) patients comprised the largest proportion, followed by triage level IV (22.1%), triage level II (8.4%), triage level I (3.5%), and triage level V (2.2%). The median daily ED census was 438 patients (IQR = 71, maximum = 756, minimum = 323). Approximately 3% of the patients were transferred from another hospital. Among the discharged patients, 96% were discharged by a physician, 3.5% discharged against medical advice, 0.5% left without notice, and only 0.02% left without being seen by a physician. Detailed demographic characteristics are shown in Table 1.

**Table 1:** Patient characteristics (n = 106,206).

Variables	Descriptive		Variables	Descriptive	
Total Length of stay (hr)*	1.46	(2.07)	Patient Disposition		
Triage to physician (hr)*	0.16	(0.16)	Discharged by physician	101972	(96.0)
physician to discharge (hr)*	1.20	(2.03)	Left without noticed	509	(0.48)
Age	35.89	(25.7)	Discharged against medical advice	3702	(3.49)
Agegroup			Left without being seen	23	(0.02)
<20	34385	(32.4)	Tests required		
20–40	26614	(25.1)	EKG obtained	11429	(10.8)
40–60	23886	(22.5)	Blood test obtained	41816	(39.4)
60–80	16369	(15.4)	X-ray obtained	47490	(44.7)
>80	4952	(4.66)	CT obtained	6868	(6.47)
Male Sex	56150	(52.9)	Consultation provided	20972	(19.8)
On critical status	392	(0.37)	Arrival time		
Transferred patients	3151	(2.97)	Day shift (08–16)	39265	(37.0)
Patient entity			Middle shift (16–24)	43460	(40.9)
Adult non-trauma	59171	(55.7)	Night shift (24–08)	23481	(22.1)
Trauma	20584	(19.4)	Weekday		
Pediatric non-trauma	26451	(24.9)	Monday	15241	(14.4)
Triage level			Tuesday	14604	(13.8)
level 1	3764	(3.54)	Wednesday	13664	(12.9)
Level 2	8919	(8.40)	Thursday	13805	(13.0)
Level 3	67676	(63.7)	Friday	13951	(13.1)
Level 4	23506	(22.1)	Saturday	15267	(14.4)
Level 5	2341	(2.20)	Sunday	19674	(18.5)
Daily ED census *	438	(72.0)			

* Presented as median (IQR)

### Survival curves for LOS of discharged patients

**Fig 1:**
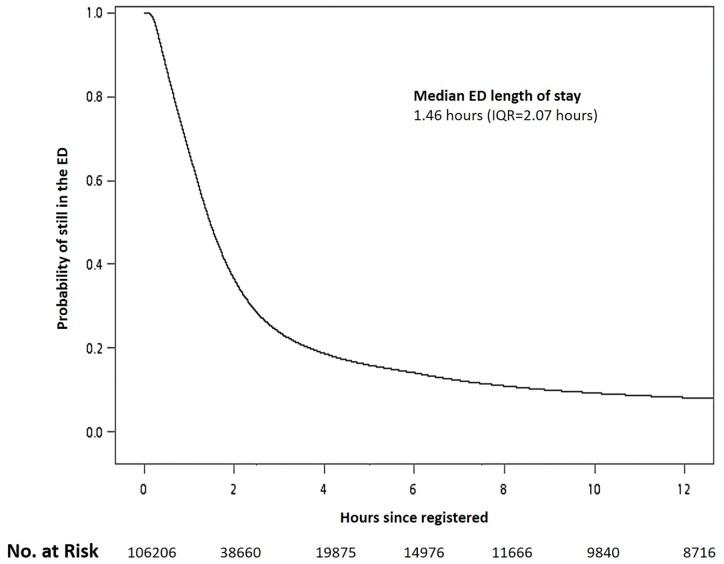
Kaplain-Meier plot for ED length of stay in discharged patients.

**Fig 2:**
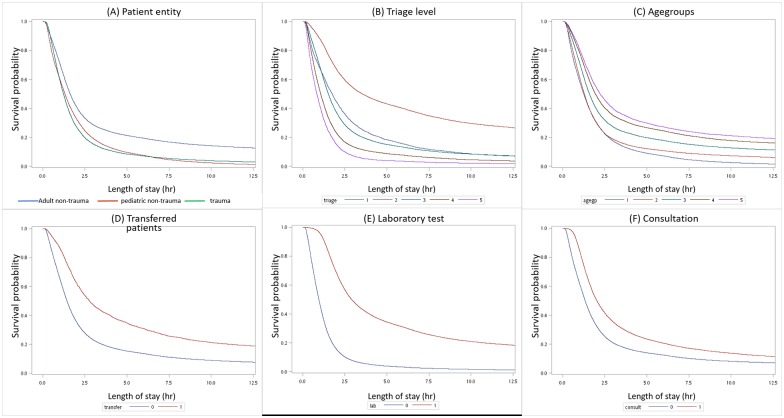
Divided Kaplain-Meier plots. The plots were divided by (a) patient entity, (b) triage level (TTAS), (c) agegroup, (d) transferal, (e) laboratory test, and (f) consultation. All showed statistical difference between stratum.

The median LOS for all discharged patients was 1.46 hours (IQR = 2.07 hours), and the median times of triage to physician and physician to discharge were 0.16 hours (IQR = 0.16 hours) and 1.20 hours (IQR = 2.03 hours), respectively. The Kaplain–Meier curve of the overall survival rate is plotted in Fig 1. The probability of being still in the ED after 1 hour, 3 hours, 6 hours, and 12 hours were 66.7%, 23.7%, 14.1%, and 8.2%, respectively. More than 90% of the included patients were discharged within 10 hours of arriving at the ED. Six additional curves stratified by patient category, triage level, age group, transferal, laboratory test, and consultation are plotted in Fig 2. All six plots showed a significant difference between strata, with *p* values <0.0001 using the log-rank test.

### Multivariate AFT analysis for factor influences and LOS prediction

**Fig 3:**
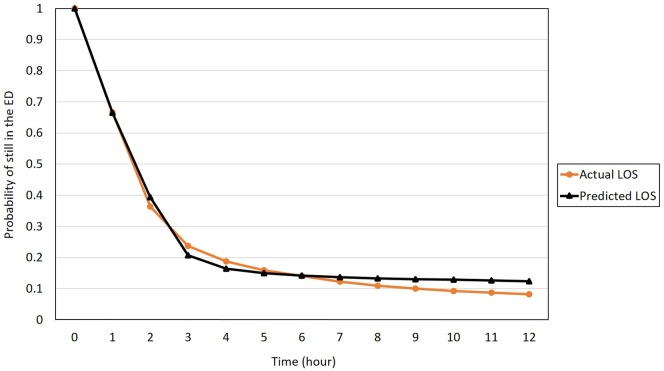
Survival curve of model predicted LOS against actual LOS.

In the multivariate AFT model, a Weibull distribution of the survival time was determined after comparing goodness of fit using the likelihood–ratio statistic between models. The results showed that increased age (>80 vs <20, TR = 1.408; 60–80 vs <20, TR = 1.432, both p<0.0001), higher acuity level (triage level I vs. level V, TR = 1.343; level II vs. level V, TR = 1.474; both p<0.0001), transferred patients (TR = 1.350, p<0.0001), obtained X-rays (TR = 1.181, p<0.0001), the patients obtaining CTs (TR = 1.515, p<0.0001) or laboratory tests (TR = 2.654, p<0.0001), a consultation provided (TR = 1.631, p<0.0001), an observation provided (TR = 8.435, p<0.0001), critical condition declared (TR = 1.205, p<0.0001), and day-shift arrival (TR = 1.223, p<0.0001) were associated with prolonged ED LOS. On the other hand, male sex (TR = 0.982, p = 0.002), weekend arrival (TR = 0.928, p<0.0001), whether the patient obtained an EKG (TR = 0.850, p<0.0001), and adult non-trauma patients (compared with pediatric non-trauma, TR = 0.687, p<0.0001) were associated with shortened ED LOS. An increased ED daily census resulted in a slight but significant increase in ED LOS (TR = 1.057, p<0.0001). Detailed results of the multivariate analyses are shown in Table 2. Using these results, individual LOS was calculated based on personal influential factors. In Fig 3 the survival curve of predicted LOS was plotted against that of observed LOS. A goodness of fit test between two curves showed lack of statistical significance (*p* = 0.649), which suggests a good predictive validity of this AFT model in the study ED.

**Table 2:** Results of multivariate accelerated failure time (AFT) model (n = 106,206).

Variables	β	95% CI of β		Time ratio (*e*^β^)	Pr > ChiSq
Agegroup					
>80	0.343	0.308	0.377	1.408	<.0001
60–80	0.359	0.333	0.386	1.432	<.0001
40–60	0.291	0.266	0.316	1.337	<.0001
20–40	0.160	0.136	0.184	1.173	<.0001
<20	0.000	.	.	1.000	.
Male Sex	-0.018	-0.029	-0.007	0.982	0.002
Specialty					
Adult non-trauma	-0.375	-0.402	-0.349	0.687	<.0001
Trauma	0.098	0.073	0.124	1.103	<.0001
Pediatric non-trauma	0.000	.	.	1.000	.
Triage Level					
Level 1	0.295	0.246	0.344	1.343	<.0001
Level 2	0.388	0.345	0.431	1.474	<.0001
Level 3	0.161	0.123	0.199	1.175	<.0001
Level 4	0.046	0.007	0.086	1.047	0.022
Level 5	0.000	.	.	1.000	.
Transferred patients	0.300	0.267	0.333	1.350	<.0001
ECG obtained	-0.163	-0.183	-0.143	0.850	<.0001
X-ray obtained	0.166	0.154	0.178	1.181	<.0001
CT obtained	0.415	0.392	0.439	1.515	<.0001
Laboratory test provided	0.976	0.963	0.989	2.654	<.0001
Consultation provided	0.489	0.475	0.504	1.631	<.0001
Admission/Observation provided	2.132	2.113	2.152	8.435	<.0001
Critical condition	0.186	0.093	0.280	1.205	<.0001
Weekend arrival	-0.074	-0.086	-0.062	0.928	<.0001
ED daily census > 95 percentile	0.056	0.029	0.083	1.057	<.0001
Arriving time					
Day shift	0.201	0.186	0.216	1.223	
Evening shift	0.143	0.129	0.158	1.154	<.0001
Night shift	0.000	.	.	1.000	.

## Discussion

In this study, we identified and quantified several influential demographic factors for the ED LOS of discharged patients, including age, patient category, triage acuity level, gender, arrival time, and transfer status. Prediction model was also made according to the analytic results, which showed a good consistency with the original observed data. In managing non-critical or non-emergency cases in the ED, most of the factors incorporated in this study—such as triage level, age, arrival time, or whether tests or consultations are provided—can be accurately obtained or anticipated to a certain extent by an experienced ED physician after the first evaluation. Although the TRs may be hospital-dependent according to the local patient population or staffing policies, the model can be used to generate information for an individualized, predicted ED LOS at a very early stage in the ED visit. This information is especially useful for patients with lower acuity, such as those managed in the LCGMH ED through the fast-track system, because these patients often complain about their waiting times. Further prospective validation is needed to test the predictive power of this model.

Some of the patient-related influential factors on ED LOS were comparable to other published reports. In a large-scale retrospective study in France, patients with lower acuity (categorized using the French clinical classification of emergency patients) were associated with a shorter ED LOS [19]. In two other studies, one using the Australian Triage System and one using the Canadian Triage Acuity System, a longer ED LOS was found in higher acuity patients [20–22]. Besides triage acuity level, advanced age has also been found to be related to a longer LOS among discharged patients [19, 23, 24]. In our study, after controlling for the effect of disease acuity, exams, or whether a consultation was provided, the effect of age group still showed a significant trend in time ratios.

Another group of influential factors includes the diagnostic activities provided in the ED. Some of the covariates, such as consultations, laboratory tests, CT exams, and radiographs have previously been mentioned [14, 15, 25, 26]. Despite the fact that more exams indicate longer ED LOS, the magnitude of the impact of these exams and services has rarely been quantified and compared. In our study, the most prominent influential factor was providing admission to the hospital or observation, followed by providing laboratory tests. Once the patients were moved to the observation area, the interval between each physician’s re-evaluation dramatically increased from minutes to hours. While some of these patients were waiting for admission and could be re-evaluated the next day, others were just receiving observation or temporary treatment. A protocolled re-assessment time may be needed to shorten the LOS of patients under observation.

Previous studies addressing multivariate event-time analysis tended to use Cox’s proportional hazard regression, but in the current study, the authors used the AFT model for two reasons. First, a Cox regression builds its model upon hazard functions, yielding hazard ratios between groups. In the current study, a hazard ratio may be interpreted as the probability that a patient in the ED with a certain factor would leave the ED in the upcoming time frame compared to that of a patient without this factor. Conversely, an AFT model builds directly upon survival time, yielding a TR. The interpretation of a TR is simply the ratio of LOS between patients with and without the factor. From the authors’ perspective, the latter is more intuitive and applicable in a clinical setting. Second, a Cox regression model addresses the hazard ratio between groups, and the baseline hazard is cancelled out during the estimation. However, to make predictions, baseline hazards need to be obtained accurately. The estimation of the baseline hazard function in the presence of ties may be problematic and requires certain types of estimator approximation [27].

One of the differences in the Taiwanese ED patient management process compared to many other countries is that patients are usually seen by a physician shortly after triage. Because of the unique social environment and easy medical access, our patients do not wait a long time before being seen by an emergency physician, as indicated by a median time of 11 minutes between triage and physician and the rate of those leaving without being seen remaining low at 0.02%. However, a very small waiting room results in more crowded queues in the treatment and observation areas. The influence of different practice behaviors on ED LOS or ED crowding may need further research to clarify.

## Limitations

There are several limitations in this study. First, it is a single-center study, and so the findings of the current research may not be generalizable or applicable in different patient populations. Second, there are still some possible correlates that are not included in the model due to the limitation of our dataset. Third, some of the discharged patients were initially arranged for admission. They were treated in the ED for one or several days while waiting for a floor bed and were then discharged by the ED physician due to their improved condition. The pattern and influential factors of the ED LOS for this group may need further analysis in a future study.

## Conclusion

This study demonstrated the influential factors of ED LOS in patients discharged from the ED. Age was associated with a progressive increase in LOS, and patients with higher acuity stayed in the ED longer. Diagnostic activities provided in the ED also had various effects on the ED LOS; among these, providing observation or laboratory tests had the largest impact. Patients who arrived during the day or on a very busy day also had a longer ED LOS. The model’s predicted ED LOS may provide useful information for physicians or patients to better anticipate an individual’s LOS and assist the administrative level in planning its staffing policy in order to improve the patient care process.

## Abbreviations

ED: emergency department
IQR: interquartile range
LOS: length of stay
LCGMH: Linkou Chang-Gung Memorial Hospital
SD: standard deviation
